# The intergluteal cleft lesion in hidradenitis suppurativa: clinical characteristics and potential clinical implications^؆^

**DOI:** 10.1016/j.abd.2026.501402

**Published:** 2026-06-27

**Authors:** Luciana Vilela Gomide, Ariany Tomaz de Aquino Saran Denofre, Juliana Yumi Massuda Serrano, Renata Ferreira Magalhães

**Affiliations:** a Department of Dermatology, Faculdade de Ciências Médicas, Universidade Estadual de Campinas, Campinas, SP, Brazil; b Department of Tropical Medicine and Dermatology, Instituto de Patologia Tropical e Saúde Pública, Universidade Federal de Goiás, Goiânia, GO, Brazil

*Dear Editor,*

Hidradenitis suppurativa (HS) is a chronic inflammatory disease characterized by painful nodules, abscesses, and draining tunnels affecting apocrine gland-bearing regions, often resulting in scarring and substantial impairment in quality of life.^1^, ^2^ Although genotype-based stratification could theoretically inform individualized prognosis, the lack of validated biomarkers and inconsistent genotype-phenotype correlations limit its applicability in daily practice. Clinically defined phenotype-based assessment, therefore, remains the most pragmatic strategy for evaluating severity and guiding treatment.^3^ Several phenotypic frameworks have been proposed, including anatomical clustering and lesion-based or comorbidity-driven subtypes; however, consensus is lacking, and underrecognized topographic patterns may provide clinically meaningful insights.^3^, ^4^, ^5^

Among these, the intergluteal cleft lesion (ICL), a midline fissure or ulceration with fibrosis, scarring, and drainage (Fig. 1), is one such pattern, infrequently described in the literature and often misclassified as pilonidal sinus disease (PSD). Given the established association between hidradenitis suppurativa and inflammatory bowel disease (IBD), particularly Crohn’s disease (CD),^6^ we sought to explore whether the presence of ICL has clinical relevance in this context.

**Fig. 1:**
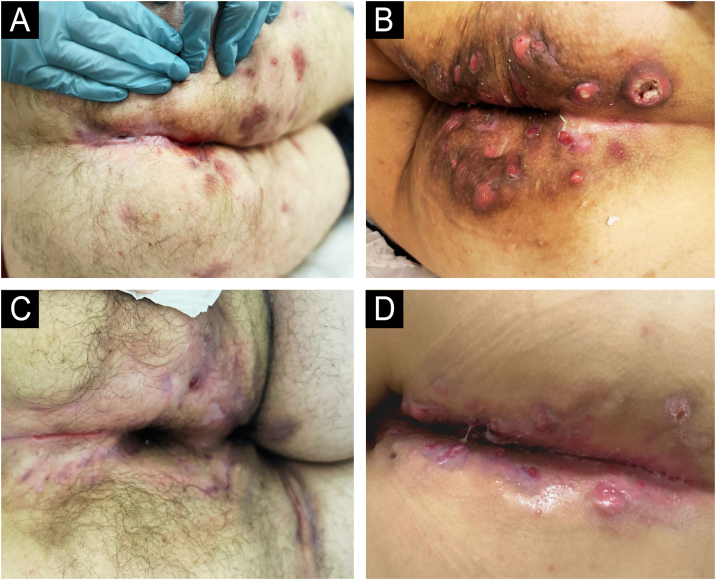
(A) Linear ulceration with surrounding inflammation in a patient with Hurley stage III HS and extensive gluteal involvement. (B) Nodular and abscessed lesions within the intergluteal cleft, consistent with severe inflammatory activity. (C) Fibrotic scarring and fissuring along the midline, suggestive of chronic disease and structural remodeling. (D) Superficial ulceration and papules confined to the intergluteal fold, in a patient without other follicular occlusion disorders.

We conducted a cross-sectional observational study to evaluate whether ICL represents a distinct clinical pattern associated with more severe HS or systemic involvement. We assessed 119 consecutive patients with confirmed HS at a tertiary dermatology center in Brazil. Standardized evaluation included demographic and clinical data, comorbidities, disease duration, number of affected regions, history of HS-related hospitalizations and surgeries, and systemic therapies. Disease activity was evaluated using the Hurley system and the International HS Severity Score System (IHS4). In 10 ICL cases, punch biopsies were performed for histopathological characterization. Comparative analyses were conducted between patients with and without ICL, and logistic regression identified independent predictors of ICL and of poor outcomes, defined as the presence of ≥ 2 of the following: Hurley III, IHS4 ≥ 11, hospitalization due to HS, or need for multiple systemic therapies.

ICL was observed in 18 of 119 patients (15.1%). Patients with ICL were more frequently male (72.2% vs. 30.7%; p = 0.002) and exhibited a markedly higher prevalence of CD (16.7% vs. 2.0%; p = 0.024) (Table 1). Disease duration and BMI did not differ significantly between groups. However, ICL was associated with substantially higher disease burden: mean IHS4 scores were more than double those of patients without ICL (16.0 vs. 7.03; p < 0.001), draining tunnels were more common (88.9% vs. 50.5%; p = 0.006), and the number of affected anatomical regions was greater (3.39 vs 2.34; p < 0.001) (Table 2). Patients with ICL also required biologic therapy more frequently and had significantly more HS-related hospitalizations in the preceding five years (55.6% vs. 12.0%; p < 0.001). Although Hurley stage III was numerically more prevalent, the lack of statistical significance suggests that ICL identifies severity dimensions not fully captured by traditional staging.

**Table 1:** Clinical and demographic characteristics of patients with and without intergluteal cleft lesions.

Variable	Intergluteal cleft lesion		p-value	Total (n)
	No (*n* = 101)	Yes (*n* = 18)		
Age, years, mean (SD)	32.8 (13.7)	34.5 (14.3)	0.650	119
BMI, kg/m^2^, mean (SD)	32.1 (6.6)	30.4 (8.4)	0.191	119
Sex, n (%):			**0.002**	119
Female	70 (69.3%)	5 (27.8%)		
Male	31 (30.7%)	13 (72.2%)		
Smoking status, *n* (%):			0.150	119
Former smoker	14 (13.9%)	2 (11.1%)		
Passive smoker	16 (15.8%)	1 (5.56%)		
Current smoker	19 (18.8%)	8 (44.4%)		
No	52 (51.5%)	7 (38.9%)		
Follicular oclusion, n (%):			0.493	119
Absent	67 (66.3%)	14 (77.8%)		
Present	34 (33.7%)	4 (22.2%)		
Crohn’s disease, n (%):			**0.024**	119
Absent	99 (98.0%)	15 (83.3%)		
Present	2 (1.98%)	3 (16.7%)		

All binary clinical variables are expressed as “Present” or “Absent”. Percentages are calculated based on the total number of patients in each group. Data are presented as mean (standard deviation), median [range], or number (percentage), as appropriate. Comparisons were performed using Mann-Whitney *U*-test for continuous variables and Chi-Square test for categorical variables. BMI, Body Mass Index.

**Table 2:** Disease severity markers in patients with and without intergluteal cleft lesion.

Variable	Intergluteal cleft lesion		p-value	Total (n)
	No (*n* = 101)	Yes (*n* = 18)		
Disease duration, years, mean (SD)	10.3 (8.58)	9.11 (5.99)	0.470	119
Number of biologic therapies received, mean (SD)	0.63 (0.60)	1.06 (0.73)	**0.030**	119
Number of affected anatomical sites, mean (SD)	2.34 (0.89)	3.39 (0.98)	**<0.001**	119
IHS4 score^a^, mean (SD)	7.03 (6.55)	16.0 (7.32)	**<0.001**	97
Hurley stage, *n* (%):			0.071	119
2	8 (7.92%)	0 (0.00%)		
3	24 (23.8%)	1 (5.56%)		
Inflammatory nodules, *n* (%):			0.054	119
Absent	69 (68.3%)	17 (94.4%)		
Present	38 (37.6%)	2 (11.1%)		
Abscesses, *n* (%):			**<0.001**	119
Absent	63 (62.4%)	16 (88.9%)		
Present	93 (92.1%)	10 (55.6%)		
Draining tunnels, *n* (%):			**0.006**	119
Absent	8 (7.92%)	8 (44.4%)		
Present	50 (49.5%)	2 (11.1%)		
IHS4 severity, *n* (%):			0.189	119
0‒3 (mild)	51 (50.5%)	16 (88.9%)		
4‒10 (moderate)	21 (20.8%)	1 (5.56%)		
≥11 (severe)	80 (79.2%)	17 (94.4%)		
Current biologic therapy, *n* (%):			0.064	119
Adalimumab	48 (47.5%)	11 (61.1%)		
Infliximab	1 (0.99%)	1 (5.56%)		
Secuquinumab	48 (47.5%)	4 (22.2%)		
No	4 (3.96%)	2 (11.1%)		
Hospitalization (past 5-years), *n* (%):			**<0.001**	118
Absent	88 (88.0%)	8 (44.4%)		
Present	12 (12.0%)	10 (55.6%)		
HS-related surgeries, *n* (%):			0.150	119
Absent	31 (30.7%)	2 (11.1%)		
Present	70 (69.3%)	16 (88.9%)		

All binary clinical variables are expressed as “Present” or “Absent”. Data are presented as mean (standard deviation) or number (percentage), unless otherwise indicated. Comparisons were performed using Mann-Whitney *U*-test for continuous variables and Chi-Square test for categorical variables.

IHS4, International Hidradenitis Suppurativa Severity Score System.

a The IHS4 was calculated using the formula: (number of nodules) × 1+ (number of abscesses) × 2+ (number of draining tunnels [fistulae/sinuses]) × 4.

Histopathological examination demonstrated chronic inflammation, granulation tissue, and fibrosis, without epithelialized tracts or embedded hairs typical of PSD, and without granulomatous inflammation suggestive of CD. Anaerobic or fungal growths were occasionally observed and likely reflected secondary colonization. Clinically, most ICLs were refractory: 66.7% exhibited only partial improvement, and 27.8% no improvement despite systemic therapy, predominantly adalimumab.

Multivariate analysis identified male sex (OR = 11.09; p < 0.001), CD (OR = 66.41; p = 0.004), older age (OR = 1.04 per year; p = 0.032), and HS-related surgery (OR = 14.52; p = 0.031) as independent predictors of ICL. Conversely, the presence of follicular occlusion syndromes was associated with lower odds (OR = 0.23; p = 0.041), suggesting partial divergence from follicular-occlusion-dominant phenotypes (Table 3). Importantly, ICL conferred an eightfold higher likelihood of poor clinical outcomes (OR = 8.37; p = 0.043), even after adjustment for demographic and clinical variables.

**Table 3:** Multivariate logistic regression: Independent predictors of intergluteal cleft lesion.

Variable	Univariate analysis				Multivariate analysis			
	OR	95% CI (lower)	95% CI (upper)	p-value	OR	95% CI (lower)	95% CI (upper)	p-value
Age, years	1.01	0.97	1.05	0.654	1.04	1.00	1.08	**0.032**
Sex:								
Male	6.07	2.09	20.32	**0.002**	11.09	3.18	47.40	**<0.001**
Crohn’s disease:								
Present	9.80	1.51	79.23	**0.017**	66.41	4.51	1975	**0.004**
Follicular oclusion:								
Present	0.58	0.16	1.77	0.368	0.23	0.05	0.86	**0.041**
HS-related surgeries:								
Present	3.43	0.90	22.54	0.115	14.52	2.02	370.61	**0.031**

Data are presented as Odds Ratios (OR) with 95% Confidence Intervals (CI), based on univariate and multivariate logistic regression models. Variables with *p* < 0.10 in univariate analysis were included in the multivariate model.

HS, Hidradenitis Suppurativa; OR, Odds Ratio; CI, Confidence Interval.

These findings identify ICL as a potential clinical marker of disease severity in HS, associated with higher inflammatory burden, adverse outcomes, and systemic involvement. HS is a heterogeneous disorder with diverse clinical endotypes, and clinically defined phenotype-based stratification has gained emphasis as a tool to identify patients at risk for refractory disease or complications.^4^, ^5^ Our results align with existing gluteal-focused clinical phenotypes described in the literature, such as the “LC3” subtype described by Canoui-Poitrine et al.^7^ and the “fistulous” variant described by Riera-Martí,^8^ both characterized by draining tunnels and aggressive behavior. The association between ICL and the absence of follicular occlusion syndromes suggests a more inflammatory pattern, less responsive to keratinization-directed therapies.

The consistently higher severity markers observed in ICL ‒ elevated IHS4, tunnel predominance, increased healthcare utilization ‒ support its role as a visible marker of advanced and treatment-resistant HS. Tunneling, a frequent feature of ICL, has previously been linked to chronicity and refractoriness, reinforcing the need for early recognition before irreversible fibrosis develops.^9^

A striking observation in this study was the strong and independent association between ICL and CD. Given the well-established coexistence of HS and IBD,^6^ this study aimed to evaluate whether the presence of ICL is clinically associated with CD. The disproportionate prevalence of CD among patients with ICL suggests that this lesion may function as a clinical marker associated with systemic inflammatory burden in HS. Given the clinical challenges in distinguishing perianal CD from gluteal HS,^10^ recognition of ICL may support earlier gastrointestinal evaluation.

Therapeutic refractoriness further underscores the need for targeted and timely interventions, potentially beyond current anti-TNF strategies, as suggested by isolated reports of IL-17 pathway efficacy in gluteal disease.^11^

This study has limitations, including a small sample size, single-center recruitment, and a cross-sectional design that precludes determining whether ICL precedes or results from severe HS. Nonetheless, the magnitude and coherence of the associations observed support the clinical relevance of this understudied lesion.

In conclusion, ICL appears to be a readily identifiable, high-yield clinical marker associated with a severe and potentially systemic form of HS. Its recognition may prompt earlier therapeutic escalation, multidisciplinary management, and targeted screening for IBD. As precision dermatology advances in HS, incorporating underrecognized clinical clues such as ICL may improve prognostication and optimize patient care.

## Authors' contributions

Luciana Vilela Gomide: Study conception and planning; preparation and writing of the manuscript; manuscript critical review; intellectual participation in propaedeutic and/or therapeutic management of studied cases; critical literature review, approval of the final version of the manuscript.

Ariany Tomaz de Aquino Saran Denofre: Manuscript critical review; critical literature review; approval of the final version of the manuscript.

Juliana Yumi Massuda Serrano: Manuscript critical review; critical literature review; approval of the final version of the manuscript.

Renata Ferreira Magalhães: Study conception and planning; manuscript critical review; intellectual participation in propaedeutic and/or therapeutic management of studied cases; approval of the final version of the manuscript.

## Ethics statement

The patients in this manuscript have given written informed consent to the publication of their case details. This study was reviewed and approved by the Research Ethics Committee of the State University of Campinas (UNICAMP) on March 26 (2024), under approval number 77223323.1.0000.5404.

## Financial support

None declared.

## Research data availability

The entire dataset supporting the results of this study was published in this article.

## Conflicts of interest

None declared.
